# Evolution of left–right asymmetry in the sensory system and foraging behavior during adaptation to food-sparse cave environments

**DOI:** 10.1186/s12915-022-01501-1

**Published:** 2022-12-27

**Authors:** Vânia Filipa Lima Fernandes, Yannik Glaser, Motoko Iwashita, Masato Yoshizawa

**Affiliations:** 1 School of Life Sciences, University of Hawai‘i at Mānoa, Honolulu, Hawai‘i USA; 2 Department of Information and Computer Sciences, University of Hawai‘i at Mānoa, Honolulu, Hawai‘i USA

**Keywords:** Laterality, VAB, Foraging behavior, Lateral line, Evolution, Cavefish

## Abstract

**Background:**

Laterality in relation to behavior and sensory systems is found commonly in a variety of animal taxa. Despite the advantages conferred by laterality (e.g., the startle response and complex motor activities), little is known about the evolution of laterality and its plasticity in response to ecological demands. In the present study, a comparative study model, the Mexican tetra (*Astyanax mexicanus*), composed of two morphotypes, i.e., riverine surface fish and cave-dwelling cavefish, was used to address the relationship between environment and laterality.

**Results:**

The use of a machine learning-based fish posture detection system and sensory ablation revealed that the left cranial lateral line significantly supports one type of foraging behavior, i.e., vibration attraction behavior, in one cave population. Additionally, left–right asymmetric approaches toward a vibrating rod became symmetrical after fasting in one cave population but not in the other populations.

**Conclusion:**

Based on these findings, we propose a model explaining how the observed sensory laterality and behavioral shift could help adaptation in terms of the tradeoff in energy gain and loss during foraging according to differences in food availability among caves.

**Supplementary Information:**

The online version contains supplementary material available at 10.1186/s12915-022-01501-1.

## Background

Laterality, commonly observed among various animal taxa, is expressed as the asymmetrical use of the left or right hand or foot [1], the biased use of the left or right eye, and the use of the left or right brain hemispheres for different processing tasks [2–9]. Laterality confers several advantages in recognizing and evading predators [10–12], facilitating complex and intricate motor activities [13] and enabling spatial learning [14]. Phylogenetic studies have shown that left–right (L–R) asymmetric traits have repeatedly and independently evolved among animals from different taxa [15], which may be attributable to the need for laterality in response to diverse environments. However, the influence of environmental factors on laterality variation and plasticity requires further elucidation—a few studies of the L-R asymmetric usage of brain hemispheres is advantageous for sound processing in bats [16, 17].

As an entry point to solve this question, we explored the environment–laterality relationship in *Astyanax mexicanus*, the Mexican tetra, which has two interfertile morphotypes, i.e., surface fish and cavefish [18–21], and is known as a model for studying the evolution of behavior. Cavefish, the blind cave-dwelling form of *A. mexicanus*, were separated from their surface-dwelling ancestors approximately 2000–20,000 years ago [22, 23]. In total, 32 *A. mexicanus* cave populations have been described in Mexico [24], and many of these populations have evolved morphological, physiological, and behavioral traits independently, likely via adaptation [21, 25–28]. One type of laterality has been reported in the Pachón cave population of *A. mexicanus* cavefish, i.e., a preference for using the right side of the body to detect novel immotile objects in the environment [29]. However, it is not yet known which environmental factors (such as perpetual darkness and prey availability) determine this right preference. Therefore, its ecological relevance remains unclear. Moreover, the evolutionary path of this right preference is unknown as such right laterality has not been investigated in ancestor-like surface fish.

In the present study, we investigated a well-characterized foraging behavior, vibration attraction behavior (VAB), which is evoked in response to 35–40 Hz vibrations [30] within the range potentially produced by soil crustaceans and percolating water [31, 32]. VAB is evolutionarily enhanced in cavefish, and its advantage was demonstrated as they hunt small prey in the dark [30]. Different degrees of increased VAB levels have been reported in lab cave populations and in the original wild cave populations [30, 33], likely owing to differences in ecological factors in these caves. Outside of cave environments, VAB may be detrimental because of nocturnal predators, such as prawn fish [34, 35].

VAB is mediated by the mechanosensory lateral line system, which is composed of sensory units known as superficial neuromasts (SNs) particularly located at the eye orbit and the third infraorbital bone (IO3) [30, 36]. The sensitivity of the SN in cavefish (Pachón) was indeed estimated much higher than that of surface fish, particularly at low frequencies (10–50 Hz) [37]. We previously reported that, despite the absence of any detectable L–R asymmetry in the SN number in surface fish and Pachón cavefish when analyzing each population “group” (i.e., within the surface fish and Pachón cavefish populations), with the SN number variations among individuals, a positive correlation could be detected between the left SN numbers and the VAB level in Pachón cavefish [38]. This asymmetric sensory–behavior correlation (hereafter termed “sensory laterality”) was not observed in surface fish at all, suggesting that this sensory laterality emerged through an evolutionary process that conferred some advantage to fish that colonized cave environments [38].

In the current study, we investigated the asymmetric L–R responses to a vibrating stimulus in *A. mexicanus* populations. In contrast to the Pachón cavefish, independent cavefish populations from the Los Sabinos and Tinaja caves did not show detectable correlations between the left or right SN number and VAB levels, indicating that asymmetric sensory–behavior linkage could be uniquely evolved in the Pachón cave. SN ablation revealed that the left SN in Pachón cavefish indeed regulate the VAB level variation. In Tinaja cavefish, we found L–R asymmetry in their approaches toward a vibrating rod that did not depend on the number of L–R SNs but was dependent on a fasting condition (i.e., depending on the internal state but not sensory inputs). Such fasting-dependent L–R plasticity was not detected in the Pachón cavefish or surface fish populations.

Our results indicate that different *A. mexicanus* cave populations have evolved distinct laterality strategies (the left sensor-based VAB observed in Pachón cavefish and the fasting-sensitive asymmetric approach seen in Tinaja cavefish), suggesting different underlying mechanisms. Based on these findings, we propose a model that explains the advantages of laterality according to diet availability and energy gain and loss. This energy-based hypothesis provides a new ground to investigate L-R asymmetry in behavior.

## Results

### Sensory laterality among different cave populations of *A. mexicanus*

**Fig. 1:**
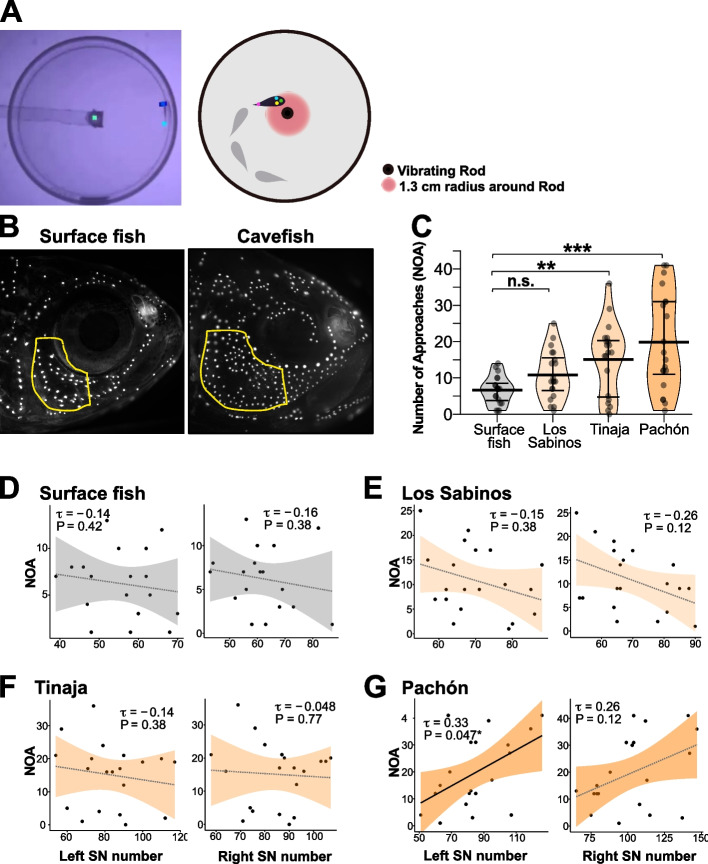
Sensory laterality in surface fish and cavefish populations of *Astyanax mexicanus*. VAB videos were analyzed using a home-developed Python script and a DeepLabCut deep learning algorithm. This method allowed us to detect the number of times fish made left or right approaches with their heads toward the vibrating rod (NOA) and the associated duration they spent within a 1.3-cm radius from the vibrating rod (DIR). **A** Video image (left) and schematic representation (right) of fish and the vibrating rod labeled with three markers on the head (left, center, and right) and one marker on the caudal fin. The left or right approaches were detected when the left or right side of the head was closer to the vibrating rod within the 1.3-cm radius. **B** Superficial neuromasts (SNs) in the infraorbital region (IO3 region delimited by a yellow line) were stained with 4-Di-1-ASP vital dye. **C** NOA data for the lab-raised populations indicated that Pachón cavefish showed the highest NOA values. The bars in the pirate plots represent means ± standard errors of the means (each population, *n* = 20). *P < 0.05; **P < 0.01; and ***P < 0.001. Statistical scores are shown in Supplementary Table 1. **D–G** The number of SNs in the left or right IO3 area plotted against the total NOA in surface fish (**D**) and Los Sabinos (**E**), Tinaja (**F**), and Pachón cavefish (**G**). Correlation analyses (**D–G**) were performed with non-parametric Kendall’s tau (*τ*). Statistical scores are available in Additional file 1: Table S2

In a previous study, we reported a positive correlation between the VAB level and the number of left- but not right-side SNs in Pachón cavefish [38] that likely evolved during the cave adaptation process. However, whether such correlation exists in other cave populations in the context of convergent/parallel evolution was unclear. Therefore, we investigated other VAB-positive cave populations, Tinaja and Los Sabinos [30, 33, 39] (Fig. 1).

First, to detect left or right approaches toward a vibrating rod, we developed a method using DeepLabCut (DLC), a machine-learning suite (Mathis et al., 2018; see the “Methods” section) [30, 39]. To validate the new method, we measured the number of approaches toward the vibrating rod (NOA) and the duration around the vibrating rod (DIR) in one surface fish and three cavefish populations (see the “Methods” section). Consistent with previous results [30, 39, 40], Pachón cavefish showed the highest level of VAB in comparison to Los Sabinos and Tinaja cavefish and surface fish, both in terms of NOA and DIR (Fig. 1C; Additional file 1: Table S1), indicating that the new detection method is consistent with the method developed previously [30, 40, 41].

**Fig. 2:**
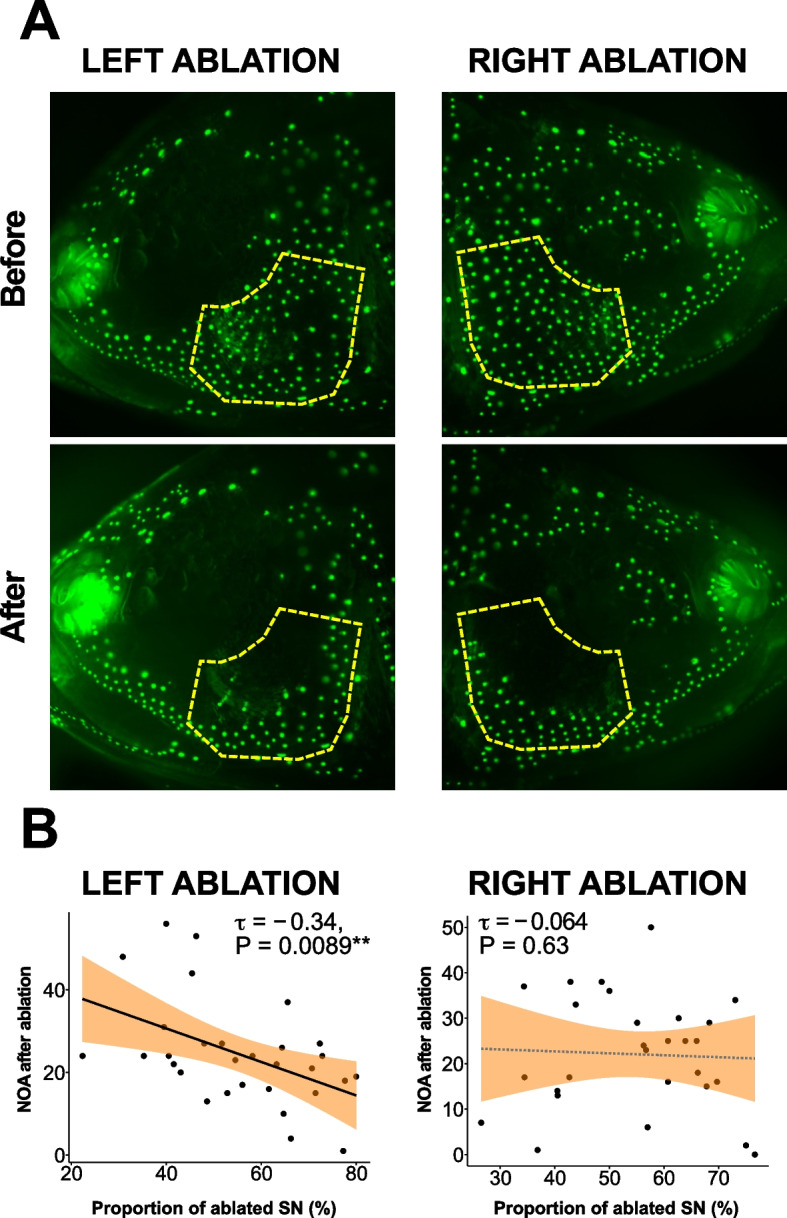
Ablation of superficial neuromasts (SNs) confirmed that the left-side SN promotes the level of VAB in Pachón cavefish. **A** Left and right sides of SNs in Pachón cavefish stained with 4-Di-1-ASP (green dots) before and after ablation of SNs in the infraorbital region (IO3 shown by a yellow line) of the same fish. **B** Proportion of ablated SNs on the left side and right side plotted against the total NOA in both cases. Regression lines with 95% confidence intervals (shaded gray or orange) are shown in each panel. See Additional file 1: Table S3 for additional details. **P < 0.01

We then explored whether an L–R bias existed in the correlations between the number of SNs and NOA or DIR. In the surface population, no correlation was detected between the left SNs and the L–R summed NOA/DIR (i.e., total NOA/DIR) or the right SNs and the total NOA/DIR (Fig. 1D; statistics in Additional file 1: Table S2; all NOA and DIR in Figs. 1 and 2 are L–R summed). In contrast, a positive correlation was found between the left SNs and NOA in the Pachón cave population (*τ* = 0.329, *P* = 0.047; Fig. 1G; Additional file 1: Table S2) but not between the right SNs and NOA (*τ* = 0.258, *P* = 0.118; Fig. 1G; Additional file 1: Table S2). This result is also consistent with our previous study [38]; thus, we tested the other cave populations with confidence. Both the Los Sabinos and Tinaja cave populations showed results similar to those of the surface population, i.e., no correlation was detected between the left SNs and NOA/ DIR or between the right SNs and NOA/ DIR (Fig. 1E, F; Additional file 1: Table S2).

In summary, our machine-learning-based detection method generated results consistent with our previous studies, showing that the left SN number is coupled with the NOA level in the Pachón cave population. However, this method failed to detect such correlations in other cave populations.

### Correlation between the left SNs and VAB is based on inputs to the left SNs

To investigate the underlying mechanism of the correlation between the left SNs and VAB, we ablated the SNs on the left or right side at the IO3 area in the Pachón cave populations using a well-developed noninvasive method [30]. To highlight SN–VAB correlation, we selected VAB-positive individuals [NOA ≥ 6; [30]]. The ablation was confirmed using DASPEI staining after the behavior assay (Fig. 2A, B). After ablation, Pachón cavefish individuals with a higher percentage of ablated SNs exhibited lower levels of VAB (*τ* =  − 0.342, *P* = 0.009; Fig. 2B; Additional file 1: Table S3). However, the change in VAB was not observed when the right-side SNs were ablated (*τ* =  − 0.064, *P* = 0.635; Fig. 2D; Additional file 1: Table S3). We also performed SN ablation in surface fish and detected no correlation between NOA and the proportion of ablated SNs on the left or right sides (Additional file 1: Figure S1 and Table S3). These results reinforced our previous findings [38] related to the significant role of the left-side SNs in the VAB of the Pachón cave population.

For DIR (i.e., the time spent located within 1.3 cm of the vibrating rod), no correlation was found with the proportions of left or right ablated SNs in Pachón cavefish (Additional file 1: Table S3), suggesting a possibility that the NOA and DIR are regulated differently, i.e., in Pachón cavefish, NOA could be mediated by the SNs, whereas DIR could be regulated via non-SN means, such as auditory and/or tactile sensing (see the “Discussion” section).

Regarding population averages, for Pachón cavefish, the ablation of the left, right, or left and right SNs induced no detectable changes in NOA or DIR (although the ablation of left-side SNs increased NOA significantly: *V* = 74.5, *P* = 0.032; Additional file 1: Figure S2 and Table S4; see also Additional file 2—extended results and discussion).

In summary, for Pachón cavefish, the left SN number raises NOA but not DIR, and the right SNs were not significantly associated with NOA.

### Behavioral laterality across different populations of *A. mexicanus*

**Fig. 3:**
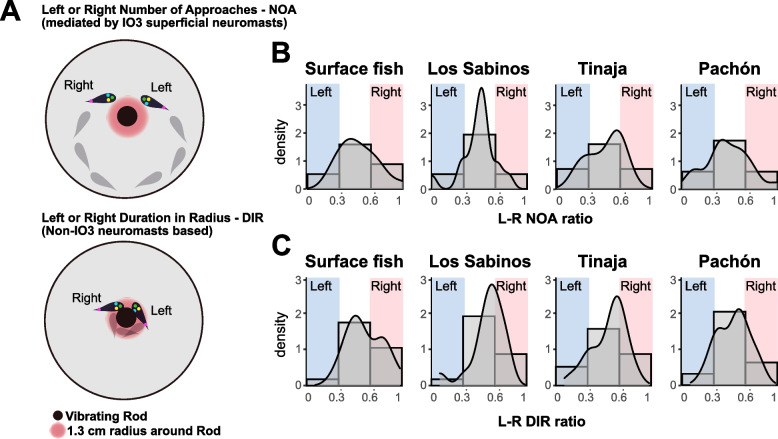
Laterality in behavioral output among surface fish and cavefish populations of *Astyanax mexicanus*. **A** Schemes representing left or right approaches expressed as NOA (top) and left or right adherence expressed as DIR (bottom). **B** Histograms for the L–R NOA ratio (the right-side NOA divided by the total NOA) across all four populations. Density curves overlaid on these data distributions. **C** Histograms for the L–R DIR ratio (the right-side DIR divided by the total DIR) across all four populations. Density curves overlaid on these data distributions. Ratios of < 0.33 indicate a strong left preference, ratios of 0.33–0.67 indicate balanced approaches, and ratios of > 0.67 indicate a strong right preference. Los Sabinos cavefish showed a gaussian-like distribution, whereas many surface fish and the other cavefish individuals exhibited strong left or right bias. Notably, all four populations included a few individuals that showed strong left or right preferences (near 0.0 or 1.0, respectively)

Our results showed that the left SNs promoted VAB in Pachón cavefish, whereas the other populations did not show L–R-biased correlation between the SNs and VAB (i.e., sensory laterality) (Figs. 1 and 2). Owing to the fact that VAB evolved as a foraging behavior and that diet type and availability differ among caves—e.g., from decomposed plants to soil or cave-adapted arthropods as well as seasonal flooding [18, 26, 30, 35, 42–44]—we hypothesized that alternative forms of laterality in VAB exist in surface and/or cave populations. Thus, we explored two types of L–R bias in VAB (i.e., left/right NOA and left/right DIR; Fig. 3A) among the surface population and Pachón, Tinaja, and Los Sabinos cave populations.

To quantify the lateral preference of individual fish, the right NOA or DIR was divided by the total NOA or total DIR, respectively (i.e., ratio scores of > 0.67 and < 0.33 indicate right and left preferences, respectively; Fig. 3B, C). We measured the L–R biases of NOA and DIR separately because our results indicated that they were likely regulated differently (see the previous section and the “Discussion” section). Individuals from each of the cave and surface populations showed a preference for one side or the other while approaching (NOA) and touching the rod (DIR) (Fig. 3B, C, respectively). This L–R preference in each individual was not repeatable or only fairly repeatable in surface fish and Pachón cavefish when we conducted assays 6 days apart and repeated three times (Table 1) [respective NOA intraclass correlation coefficients (ICCs): *κ* = 0.0 (none), *P* = 0.48; *κ* = 0.39 (fair), *P* = 0.00025. Respective DIR ICCs: *κ* = 0.0 (none), *P* = 0.48; *κ* = 0.085 (none), *P* = 0.20]. In contrast, Tinaja cavefish individuals exhibited a moderate level of repeatability in NOA (*κ* = 0.54, *P* = 0.0021; repeated three times; Table 1), suggesting that these fish tended to consistently approach the vibrating rod unidirectionally.

**Table 1:** Intraclass correlation coefficient (ICC) under Cohen’s suggestion [45, 46] (*N* = 3 repeats; *N* = 22 for surface, *N* = 9 for Pachón cave, and *N* = 11 for Tinaja cavefish)

Population	Measurement	Average L–R ratio	Statistics	Kappa	*P*-values
Surface fish	NOA	0.459 ± 0.034	*F*_21,42_ = 1.0	0.0 (none)	0.48
Surface fish	DIR	0.481 ± 0.028	*F*_21,42_ = 1.0	0.0 (none)	0.48
Pachón cavefish	NOA	0.464 ± 0.036	*F*_28,56_ = 3.0	0.39 (fair)	0.00025***
Pachón cavefish	DIR	0.473 ± 0.024	*F*_28,56_ = 1.3	0.085 (none)	0.20
Tinaja cavefish	NOA	0.482 ± 0.052	*F*_10,20_ = 4.5	0.54 (moderate)	0.0021**
Tinaja cavefish	DIR	0.478 ± 0.046	*F*_10,20_ = 2.4	0.32 (fair)	0.043*

As shown in the histograms in Fig. 3B and C, there was no detectable bias between the left and right-side NOA (*F*_1,152_ = 0.149, *P* = 0.700) or between the left and right DIR among the populations (*F*_1,152_ = 3.378, *P* = 0.068) (Additional file 1: Figure S3 and Table S1). Additionally, there was no detectable bias between the duration of left and right facing outside the 1.3-cm radius around the vibrating rod, suggesting that there was no tendency to face left or right in relation to the vibrating rod from a distance (*F*_3,152_ = 0.016, *P* = 0.997; Additional file 1: Table S1).

The above results suggested that laterality in SN usage (sensory: Pachón cavefish) and laterality in approaches (behavior: Tinaja cavefish) existed in some *A. mexicanus* populations. Therefore, we investigated whether relationships existed between the lateralities in the number of left or right SNs and left or right NOA or left or right DIR. Detailed correlation analyses were performed in the ablation study. In Pachón cavefish, the ablation ratio of the left SNs mirrored the reduction of left approaches (left NOA), but no correlation was detected between the left SN ablation ratio and the right NOA, the right SN ablation ratio and the left or right NOA, and the left or right SN ablation ratio and the left or right DIR (Additional file 1: Table S3). Additionally, there was no detectable effect of any left or right SN ablation on the L–R approaches (NOA or DIR) of surface fish. These results indicate that the number of left SNs (inputs) affects the left approaches of Pachón cavefish but not those of surface fish. As in the intact SN condition, there were some positive correlations between the number of L–R SNs and the L–R NOA and/or DIR in surface fish and Pachón cavefish (but not in other cave populations; see Additional file 1: Table S2). However, the SN–behavior relationship of surface fish in the intact state may not be vital because it was not detected in the ablation study.

Collectively, these results indicate that sensory laterality was found only in Pachón cavefish and was associated with left-side approaches. Individuals in the Tinaja cave population but not in the surface or Pachón cave populations showed a moderately consistent preference for left- or right-side approaches, and L–R bias was not detectable at the whole population level.

### Fasting increased sensory responses and reduced approach laterality

Previous prey-capture experiments indicated that VAB promotes the food capture rate in dark and food-sparse environments [30]. Many caves in North-eastern Mexico experience wet and dry seasons; thus, cavefish experience food-sparse conditions for ~ 6 months due to these seasonal changes [44]. Thus, we hypothesized that fasting induces shifts in laterality. Because younger fish are more sensitive to fasting in general, we used 1-year-old (young adult) surface fish, Pachón cavefish, and Tinaja cavefish rather than the 2–3-year-old (adult) fish used in the earlier experiments (Figs. 1, 2, and 3).

**Fig. 4:**
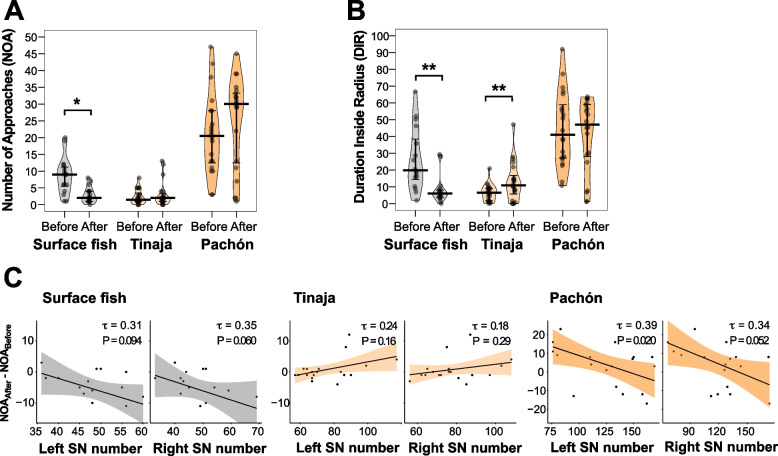
Plasticities of surface fish and Tinaja cavefish in total NOA and/or DIR, and the plastic left SN–NOA association in Pachón cavefish in response to fasting. **A** Pirate plots showing total NOA. Surface fish showed significantly reduced NOA after fasting, but neither Tinaja nor Pachón cavefish showed significant changes in NOA. **B** Pirate plots showing total DIR. Surface fish showed significantly reduced DIR after fasting, but Tinaja’s DIR increased after fasting. Pachón cavefish showed no significant changes in DIR. **C** The number of left or right SNs plotted against changes in NOA after fasting (NOA_after_ − NOA_before_) in surface fish, Tinaja cavefish, and Pachón cavefish. In Pachón cavefish, individuals with fewer left SNs showed a higher increase in total NOA, suggesting that a plastic response to fasting existed in individuals with few left SNs and a low NOA level. There was no detectable correlation between the number of right SNs and the change in NOA in Pachón cavefish or the other populations. All statistical scores are available in Additional file 1: Table S5 and S6

First, we investigated the effect of fasting on sensory laterality (i.e., the number of left or right SNs vs. the total NOA or DIR) among the surface, Pachón, and Tinaja populations. The 6-day fasting reduced the total NOA and total DIR in surface fish but increased the total DIR in Tinaja cavefish (Fig. 4A, B; Additional file 1: Table S5); however, no changes were detected in Pachón cavefish (Fig. 4A, B; Additional file 1: Table S5).

To investigate L–R SN–behavior correlation, we determined the correlations between either the number of left or right SNs and the total NOA after fasting with subtraction of the prefasting NOA (Fig. 4C; Additional file 1: Table S6). Pachón individuals with fewer “left” SNs exhibited a higher increase in total NOA (Fig. 4C; Additional file 1: Table S6). Pachón individuals with fewer “right” SNs showed a higher increase in NOA but not at a significant level (Fig. 4C; Additional file 1: Table S6). Surface fish or Tinaja cavefish did not show fasting-dependent changes in SN–approach correlation (Fig. 4C; Additional file 1: Table S6). In summary, fasting yielded different responses in the surface and Pachón populations, i.e., NOA was reduced in surface fish, and Pachón individuals with fewer left SNs showed a higher increase in NOA relative to that of individuals with more left SNs.

**Fig. 5:**
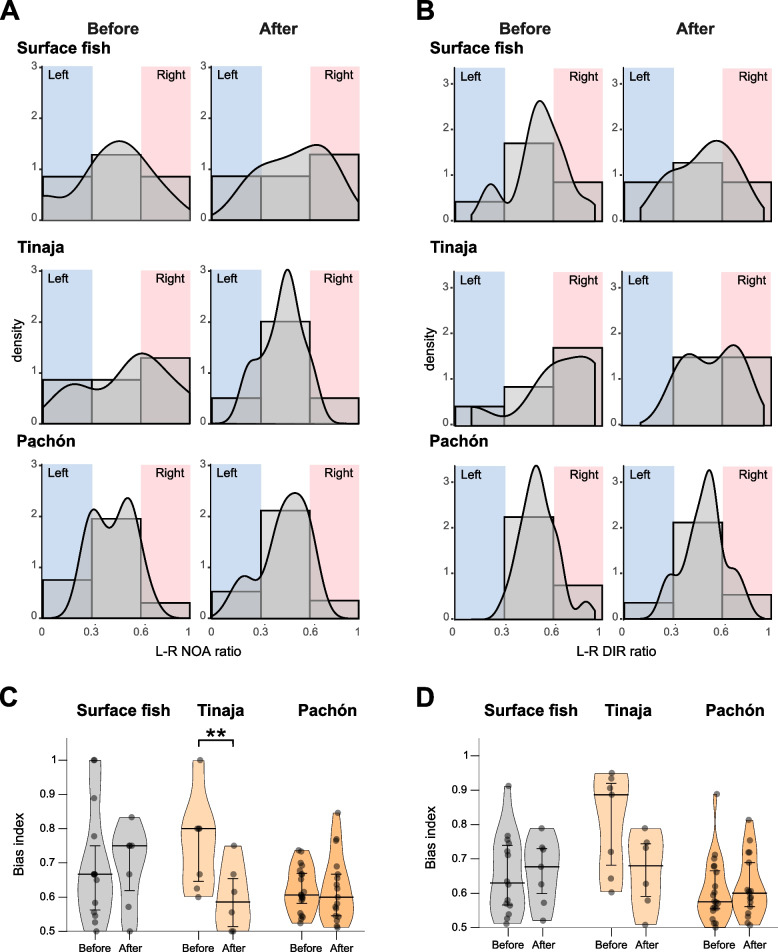
Right-biased approaches (NOA) in Tinaja cavefish were reduced after fasting. **A**, **B** Histograms and density plots showing the L–R NOA ratio (**A**) and L–R DIR ratio (**B**) before and after fasting in the indicated populations. In **A**, more Tinaja cavefish individuals showed L–R balanced approaches (0.33–0.67) after fasting; Pachón cavefish maintained L–R balanced approaches (0.33–0.67) before and after fasting, whereas surface fish showed a slight right bias. L–R NOA ratio = $\frac{\mathrm{NOA}(\mathrm{right})}{\mathrm{NOA}(\mathrm{right})+\mathrm{NOA}(\mathrm{left})}$. In **B**, the trends for DIR were similar to those in (**A**). L–R DIR ratio = $\frac{\mathrm{DIR}(\mathrm{right})}{\mathrm{DIR}(\mathrm{right})+\mathrm{DIR}(\mathrm{left})}$. **C, D** Pirate plots showing L–R bias index for NOA (**C**) and DIR (**D**). In **C**, right bias index = $\frac{\mathrm{max}\{\mathrm{NOA}\left(\mathrm{right}\right),\mathrm{ NOA}\left(\mathrm{left}\right)\}}{\mathrm{NOA}\left(\mathrm{right}\right)+\mathrm{NOA}(\mathrm{left})}$. Tinaja cavefish showed a significantly reduced bias index for NOA after fasting, indicating balanced L–R approaches, whereas the other populations showed no detectable changes. In **D**, L–R bias index = $\frac{\mathrm{max}\{\mathrm{DIR}\left(\mathrm{right}\right),\mathrm{ DIR}\left(\mathrm{left}\right)\}}{\mathrm{DIR}\left(\mathrm{right}\right)+\mathrm{DIR}(\mathrm{left})}$. None of the populations showed detectable changes in the bias index after fasting, although Tinaja cavefish showed a nonsignificant trend for reduced bias after fasting. All statistical scores are available in Additional file 1: Table S7. **P < 0.01

Second, we studied the effect of fasting on laterality in approaches. The surface and Tinaja populations had more number of younger fishes (1-year-old fish vs. 2–3-year-old fish) that showed L–R-biased approaches in the pre-fasting condition (NOA; broader standard variations; Fig. 5A, B). In contrast, Pachón cavefish individuals showed L–R symmetrical NOA with a narrower standard variation than that observed in the surface and Tinaja populations in the pre-fasting condition (Fig. 5A, B).

After 6 days of food deprivation, the distributions of the L–R preference in surface fish became more uniform for both NOA and DIR (Fig. 5A–D; Additional file 1: Table S7). Interestingly, Tinaja cavefish shifted from a right-biased approach (for both NOA and DIR) to a symmetrical approach in NOA (~ 0.6 in a bias index; Fig. 5C) and a reduced right bias in DIR (Fig. 5). Regarding population averages, none of the surface fish, Tinaja, or Pachón cavefish individuals showed detectable L–R bias in NOA or DIR in the prefasting or postfasting condition, except DIR in Tinaja cavefish (right bias; Additional file 1: Figure S4A and 1B), which mirrors the observation in Fig. 5A and B. Fasting also induces alternative responses among surface fish, Tinaja, and Pachón cavefish individuals; surface fish decreased the overall NOA and DIR after fasting, while Tinaja cavefish increased DIR (Additional file 1: Figure S4A and 1B). Pachón cavefish did not show detectable changes in the overall NOA and DIR levels.

In summary, food deprivation drove behavioral shifts in the surface and Tinaja cave population but not in the Pachón cave population, i.e., surface fish, showed reduced VAB, whereas Tinaja cavefish showed increased VAB (Fig. 5, and Additional file 1: Figure S4). Furthermore, Tinaja cavefish showed a postfasting plastic change in the L–R bias of NOA toward a more L–R symmetric approach (Fig. 5, and Additional file 1: Figure S4).

## Discussion

We addressed laterality in sensory–behavior association among different populations of *A. mexicanus*. Using a newly developed DLC- and machine learning-based video analysis method, we found the following: (1) only the Pachón cave population showed a bias in SN–VAB association, i.e., the left SNs promote VAB, but not detectable in other populations; (2) no L–R bias detected in approaches (NOA) and adherence (DIR) in the surface, Pachón, Tinaja, and Los Sabinos cave populations at the population levels, but some individuals, especially those in the Tinaja cavefish population, repeatedly showed a preference for left or right approaches; and (3) fasting for 6 days reduced the L–R-biased in approaches in the Tinaja cave population, indicating that VAB plasticity is present in this population. Thus, L–R asymmetry in foraging behavior seems to have evolved flexibly under different caves, and our findings provide insights into the evolutionary dynamics of L–R asymmetry under ecological pressure (see below). At the end, we discuss the energy-saving advantage associated with foraging laterality.

### Introducing a machine-learning method to assess adaptive foraging behavior

With the aid of a DLC neural network (trained with 807 images), we developed a new video analysis method that achieved a detection accuracy of > 99%, which was confirmed using a manual survey of the labeled videos. In comparison with our previously reported method [30], this trained network yielded higher resolution for the approach (NOA) and adherence (DIR) events of VAB, and left or right facing in the direction of the vibrating rod could be monitored. The new method was also confirmed to be consistent with the ImageJ-based method developed previously (Fig. 1), which supports the findings of the present study as an expansion of the previous results.

### Correlation between the left SNs and NOA but not DIR in Pachón cavefish

Pachón cavefish showed a significant correlation between the number of left SNs and NOA. This correlation was observed repeatedly in different cohorts from different Pachón parents [38]. Furthermore, this sensory laterality in NOA in Pachón cavefish was confirmed in our ablation experiment, i.e., as more left SNs were ablated, the NOA was reduced. Notably, although the left SN number and the NOA level were correlated, no association was observed between the left SN number and the left approach (left NOA) or the right approach (right NOA). This fact suggests that the SN left sensing only stimulates the appetite but would not inform regarding the location of the prey. We will confirm this hypothesis by imaging the activity at the appetite center with the immediate early genes signal [47] in the near future.

Furthermore, a correlation between the left SNs and DIR was not detected in Pachón cavefish. Such variation in sensory–behavior association indicated that differences exist in the sensory mechanisms underlying NOA and DIR, i.e., owing to the correlation between the SNs and NOA and our SN ablation results, NOA in Pachón cavefish is likely enhanced by SNs. Contrastingly, DIR was not correlated with the number of SNs or changed after SN ablation, which suggested that other sensory systems, e.g., tactile and/or auditory sensors, affect DIR. The auditory inner ear can sense near-field particle motion based on the motion lag between the fish body and the otolith [48–50]. Overlapping functions between auditory, lateral line, and tactile senses have been noted but not studied in detail in the context of foraging (e.g., [51, 52]). VAB thus emerges as a good behavior model of the modality switch between these three sensory systems because it can be evoked from the far-field (> 5 m in the field: auditory; see below; [33]) and near-field (possibly; > 1.3 cm: lateral line; < 1.3 cm: tactile).

### Laterality in cranial morphology and behavior

There are reports of cranial bending in adult Pachón and Tinaja cavefish but not in adult surface fish or younger individuals of the Pachón, Tinaja, and surface populations [53]. We did not detect left cranial bending in our experimental fish (2–3 cm standard length; 1–3 years old), which excludes the confounding effect of head bending on lateralities in sensory–behavior association and approaches to the rod. Other morphological asymmetries have been reported previously, e.g., L–R asymmetry in the suborbital (i.e., infraorbital) bone fragmentations in Pachón and Tinaja cavefish but not in surface fish [54], and L–R asymmetry in cranial SN distribution but not the number of SNs, which corresponds to asymmetric bone fragmentation [54, 55]. In the present study, we investigated the L–R asymmetric “number” but not “distribution” of SNs. It will be interesting to investigate whether the SN distribution pattern plays a role in VAB expression in future studies.

### Laterality of approaches at the population and individual levels, and the effect of starvation

In our L–R approach study, no detectable left or right bias was observed for NOA and DIR at the population level among the four tested populations (with the exception of 1-year-old Tinaja cavefish). However, some individual fish showed one side bias in NOA and/or DIR, and these asymmetric biases were consistent within each individual. Laterality at the individual level has been largely overlooked [56]; regardless, studies in chicks and fish showed that behavior lateralization was random in a population but existed and seemed advantageous (e.g., by minimizing the use of brain resources) at the individual level in complex tasks, including social interactions [6, 57, 58]. In the current study, we also revealed plastic laterality in a cave population responding to fasting. Because VAB evolved as a foraging behavior under food-limited conditions [30, 35], we propose that cavefish laterality evolved to conserve energy.

Many caves in North-eastern Mexico experience 6-month wet and 6-month dry seasons; thus, cavefish may experience conditions of food scarcity for ~ 6 months per year [44]. We tested the effect of food deprivation on L–R asymmetry, and removing food intake for 6 days increased both DIR and L–R symmetric NOA in the starvation-resistant Tinaja cavefish [59]. The increases in DIR and symmetric NOA could help boost food intake while saving energy (see below). In contrast, the surface fish, which are less tolerant to starvation, showed decreased NOA and DIR, seemingly saving energy in a simpler manner by reducing the number of foraging attempts. The most derived Pachón cave population [23] is also starvation resistant but showed a nonsignificant change in symmetric NOA and DIR, perhaps because these cavefish have optimized their foraging under food-sparse conditions over the course of evolution (see below). In the Additional file 2—extended results and discussion, we also discuss age-dependent L–R plasticity in Tinaja cavefish (c.f., Figs. 3B, C and 5A, B) and the VAB levels of Los Sabinos and Tinaja cavefish.

### Advantages of laterality in the cave ecosystem

To understand the evolution of laterality and its plasticity in the context of cave ecology, we considered energy intake and consumption in food-limited conditions. Generally, maximizing the total energy gain by balancing energy intake (i.e., food intake) and consumption (e.g., foraging behavior) is one of the attributes of fitness in a given environment. Accordingly, the total energy gain (i.e., foraging efficacy) is studied in the context of trade-off in many animal models [60]. In the present study, we explained that sensory laterality and symmetrical approaches are advantageous in food-limited environments owing to the tradeoff between calorie gain from food and calorie use in the foraging strategy.

As for unilateral sensing observed in Pachón cavefish, we supposed that the foraging motivation (appetite) of the fish is only evoked by one side of the sensory system in response to the vibration stimulus (from the food); therefore, foraging behavior is evoked by a half a chance compared to bilateral sensing fish. Therefore, only half of the lateral-sensing fish aim to capture the food. Once the fish’s appetite is stimulated, the fish could approach the food/vibrating source from both L-R sides (see below). In this case, lateral sensing is better especially in food-limited conditions, because fewer fish compete for the limited food and, therefore, the total energy gain exceeds the cost of approaching the food (Additional file 1: Figure S5 and Additional file 2). However for the bilateral sensing fish, many fish fail to consume the food by competing with the others, thereby losing energy on average (Additional file 1: Figure S5 and Additional file 2). In summary, we expect that the unilateral sensing population will save relatively more energy; therefore, such sensing can be considered advantageous to the food-sparse environment because of the reduced energy consumption.

As for the L–R-biased approach observed in Tinaja fish, we supposed that the biased approach to the food consumes more energy than the non-biased because, at the vicinity of the food, the individual frequently needs to turn steeply towards the food to keep using one side. This approach tendency would cost higher energy in total (Additional file 1: Figure S5 and Additional file 2). Therefore, a non-biased approach is assumed to be less energy intensive and beneficial especially in conditions of food scarcity.

Accordingly, Pachón cavefish is the most optimized for the food-sparse environment (left SN-associated VAB but no L-R bias while it approached the vibrating rod; Figs. 1, 2, and 5A, C). The degree of laterality in Pachón and Tinaja cavefish populations fits well with their ecological conditions where Pachón cavefish experience seasonal availability of animated diets, crustaceans, that is available more in the ~ 6-month dry season than in the 6-month wet season [26, 44]. In contrast, several Tinaja cave pools receive percolating water most of the time and have thick organic matter covering the bottom, distinct from Pachón cave, and the plastic laterality may be sufficient for adaptation to this cave environment [26].

## Conclusions

In summary, laterality in sensory-behavior association and symmetrical approaches may have advantages in food-sparse environments and may have promoted adaptation processes in the Pachón cave. Over the course of evolution, Tinaja cavefish evolved a plastic foraging strategy according to food availability, whereas Pachón cavefish evolved a robust SN sensory laterality with balanced approaches to vibrating objects. These results represent new insights that help us better understand the evolution of laterality, and they may lead to further insights into the diversification of laterality among animals.

## Methods

### Astyanax mexicanus husbandry and ethics statement

The lab-raised surface fish population used in this study is descended from an original collection from Balmorhea Springs State Park (TX, USA), whereas the lab-raised cave populations used in this study are descendants of an original collection from Cueva de El Pachón in Tamaulipas collected by Richard Burowski (2009/2011), and collections from Sótano de la Tinaja and Cueva de Los Sabinos in San Luis Potosí, Mexico collected by Bill Jeffery in (2000–2001). The adult fish (1–3 years old) used in the experiments were raised in groups of 20–25 individuals in 0.74-L Ziploc containers (S.C. Johnson & Son Inc., Racine, WI, USA) and 1.4-L Zebrafish Aquatic Housing System tanks (Aquaneering Inc., San Diego, CA, USA). Fish were fed twice per day ad libitum with live *Artemia salina* larvae (Brine Shrimp Direct, Ogden, UT, USA). In this study, we did not discriminate fish sex and randomly mixed in all experiments.

All animal procedures were performed under protocol 17–2560 approved by the Institutional Animal Care and Use Committee at the University of Hawai ‘i.

### Recording VAB

VAB assays were performed as described previously by Yoshizawa et al. [30] and Worsham et al. [61]. Briefly, fish were acclimated for 4–5 days in a cylindrical chamber (10 cm in diameter; 5 cm in height; Pyrex 325 mL; Corning, NY, USA) filled with conditioned water (pH 6.8–7.0; conductivity of ~ 700 μS). Fish were fed once per day ad libitum with live *A. salina* larvae. After acclimation, the behavior of fish in response to vibrations was recorded for 3 min immediately after the introduction of the vibration rod into the bowl. The vibration stimulus was generated using a 7.5-mm diameter glass rod at 40 Hz with a GW Instek SFG-1003 DDS Function Generator (Good Will Instrument Co., Ltd., New Taipei City, Taiwan) connected to an audio speaker (ProSpeakers, Apple, Cupertino, CA, USA). Video footage of behavior was recorded under infrared illumination (880-nm wavelength; BL41192-880 blacklight; Advanced Illumination, Rochester, VT, USA) using a customized Microsoft MAIN-31891 LifeCam Studio installed with a Zoom lens (Zoom 7000, Navitar, Rochester, NY) and VirtualDub2 video capture software (licensed under the GNU General Public License: https://sourceforge.net/projects/vdfiltermod/).

The NOA was analyzed using ImageJ macro plugins (ImageJ 1.53a; NIH, Bethesda, MD, USA) and a homemade script published previously [30, 61]. Using this method, we measured NOA and selected Pachón cavefish that exhibited NOA ≥ 6 for the ablation study (see the “Ablation experiments” section).

### VAB video analysis using DLC

Videos were analyzed using the DLC toolbox version 2.0.6 [62, 63] in Python 3.6. DLC provides a robust suite of tools for deep learning-based markerless pose estimation. The approach is based on transfer learning, wherein a deep neural network is initially trained on an initial task with large amounts of available training data to achieve robust initial parameterization and then fine-tuned on a target task (ideally as closely related to the initial task as possible; in this case, pose estimation) using a smaller dataset. DLC uses a ResNet50 [64] backbone pretrained on the ImageNet [65] dataset to classify 1000 object categories in a dataset of over one million hand-labeled images. This network parameterization is then transferred and fine-tuned, during which the last layer of the network used originally for classification is replaced with transposed convolutional layers for pose estimation. These new layers predict individual probability densities over all pixels of a given input image for each pose marker, and such predictions are repeated independently for each frame in the video. DLC was also used to simplify data selection, data labeling, training, evaluation, and retraining processes. In this study, we specified a pose estimation task to track both the fish and rods in the VAB videos. For the fish, three head markers were tracked (right-side, left-side, and center) as well as one marker in the caudal fin; additionally, one marker was placed in the center of the vibrating rod. Because some videos contained two Petri dishes and some contained one, two separate sets of the same markers were allocated. The initial training set included frames selected randomly from 88 different VAB videos, and 807 hand-labeled training frames (images) were used to train our final model. The ResNet50 model was fine-tuned until the DLC early-stopping criterion was reached while using the default DLC training parameters as described by Mathis et al. [62]. All weights in the network were adjusted during training. The trained model was used to predict marker locations in all videos, outputting the *x* and *y* coordinates associated with the highest probability pixel for each marker. In frames with at least one DLC marker below a predefined confidence threshold, all marker locations were interpolated based on the markers’ positions in the preceding and following frames, which ensured that fish tracking was smooth throughout the duration of each video. Specifically, for a sequence of one or more subsequent frames where each frame had at least one marker below the confidence threshold, all markers’ locations were linearly interpolated between each of the respective markers’ locations in the frame immediately preceding and the frame immediately following that sequence. For videos including multiple recording arenas, markers for each Petri dish were treated independently. The confidence threshold was set empirically to 0.92 after manual inspection of the DLC outputs. The resulting marker locations were used to track several behaviors of interest in the videos: time spent within a 1.3-cm radius of the rod (DIR), time spent being outside of this radius (DOR), left- and right-sided approaches (NOA), and total swimming distance. The 1.3-cm radius from the vibrating rod was determined as the cutoff of VAB because it was the distance that best discriminated between the surface fish and cavefish approaches across the tested radii of 0.5–2.5 cm (in 0.1-cm increments) from the vibrating rod. A left-sided approach was defined as entering the 1.3-cm radius threshold with the head-center marker while the head-left marker was closer than the head-right marker to the rod, and vice versa for a right-sided approach (Fig. 3A). Accordingly, DIR, DOR, and NOA were separated as right- or left-facing based on the proximity of the head-right and head-left markers to the rod (Fig. 3A).

### Repeatability test

Repeatability test was performed as described previously [30]. Briefly, we first acclimated 22 surface as well as 9 Pachón and 11 Tinaja cave fish in cylindrical chambers for 4–5 days. Fish were fed once per day ad libitum with live *A. salina* larvae and their movement was recorded as described in the Recording VAB section. After the recording, the cylindrical chambers were cleaned and fish were maintained in the same chambers for 6 days, then recorded again on day 7 of the second acclimation (6 days apart). This procedure was repeated for a third recording. The conditioned water was replaced every 2 days during this period. After the behavior analysis using DLC, interclass correlation repeatability (3 repeated measurements) was tested as referring Single_random_raters by the “psych” R package.

### SN imaging

Neuromast vital staining was performed as described previously [30, 61, 66]. Fish were immersed in 25 μg/mL of 4-(4-(dimethylaminostyryl) − 1-methylpyridiniumiodide (4-Di-1-ASP; MilliporeSigma, Burlington, MA) dissolved in conditioned water for 40–60 min, after which they were anesthetized in 66.7 μg/mL of ice-cold ethyl 3-aminobenzoate methanesulfonate salt (MS222; MilliporeSigma) in conditioned water (pH 6.8–7.0; conductivity of ~ 700 μS). Fish were then visualized using a fluorescence microscope [BX61WI Olympus microscope with a 2.5 × MPlanFL N lens, a Rhodamine filter set, and an ORCA-Flash 4.0 digital camera (Olympus Corp.)]. SNs were counted from digital images of 4-Di-1-ASP-stained fish using the “Analyze Particles” function of ImageJ 1.53a. SNs were counted both in the left and right sides of the third infraorbital/suborbital bone.

### Ablation experiments

Ablation experiments were performed using 1-year-old surface fish (*n* = 47) and VAB-positive cavefish (Pachón population, *n* = 67). The VAB level was measured before ablation, and VAB-positive cavefish (NOA ≥ 6 according to ImageJ-based analysis) were selected strategically to determine the association between IO3 SNs and VAB level.

SN ablations were performed using Vetbond (3 M, St. Paul, MN, USA), a nontoxic tissue adhesive, on the left (surface fish, *n* = 14; Pachón cavefish, *n* = 30), right (surface fish, *n* = 13; Pachón cavefish, *n* = 25), and both left and right (surface fish, *n* = 10; Pachón cavefish, *n* = 12) sides (Fig. 2A, B). Fish were anesthetized in 66.7 μg/mL of ice-cold ethyl 3-aminobenzoate methanesulfonate salt (MS222, MilliporeSigma) in conditioned water (pH 6.8–7.0; conductivity of ~ 700 μS). Excess water was removed carefully using Kimtech wipes, and the adhesive (0.5 μL) was applied to the skin surface. After being exposed to the air for 10 s, treated fish were placed in bowls (10 cm in diameter; 5 cm in height; Pyrex 325 mL, Corning) filled with conditioned water at room temperature. Within a day of active swimming, the adhesive usually peeled off, and VAB was assayed. 4-Di-1-ASP staining was performed to count the number of SNs in the IO3 region before and after ablation.

### Fasting

Pachón cavefish (*n* = 20), Tinaja cavefish (*n* = 20), and surface fish (*n* = 16) individuals (~ 1 year old) were placed in cylindrical chambers (10 cm in diameter; 5 cm in height; Pyrex 325 mL, Corning) and acclimated for 4–5 days in conditioned water (pH 6.8–7.0; conductivity of ~ 600 μS) in which they were fed once a day (weight or approximate number of *Artemia*) with live *A. salina* larvae. After acclimation, VAB was recorded and analyzed. Following this first assessment, fish were starved for 6 days and VAB was recorded and analyzed once again.

### Statistical analysis

All statistical analyses were performed in RStudio 4.0.3 (RStudio, Boston, MA, USA). The R packages used included lme4, lmerTest, car, coin, yarrr, AICcmodavg, and ggpubr. Linear or generalized linear models were selected using Akaike’s information criterion function to identify the best fit models for the NOA and DIR analysis in all experiments. Analysis of variance (ANOVA) was performed using generalized linear model fitting functions (glm or glmer in the lme4 package). Post hoc tests were performed using the Wilcoxon signed-rank (paired) test followed by Holm’s multiple-test correction. Nonparametric Kendall’s tau correlation was used to evaluate associations between neuromasts and behavior outputs.

## Supplementary Information

**Additional file 1: Fig. S1.** Correlation plots in SF. **Fig. S2.** Overall ablation plots in PA. **Fig. S3.** Left and Right NOA and DIR across populations. **Fig. S4.** Left and Right NOA and DIR before and after starvation. **Fig.S5.** Evolutionary model for sensory and behavior laterality. **Table S1.** Statistical Scores for Figs.1 and S3. **Table S2.** Correlation Scores for Fig. 1. **Table S3.** Correlation Scores for Fig. 2. **Table S4.** Statistical Scores for Fig.S2. **Table S5.** Statistical Scores for Figs.4 and S4. **Table S6.** Correlation Scores for Fig. 4. **Table S7.** Statistical Scores for Fig.5C and D.

**Additional file 2.** Extended results and discussion.

## Data Availability

The video datasets generated and analyzed during the current study are available at https://doi.org/10.5281/zenodo.7392181 [67]. All program scripts used in this study are available at https://doi.org/10.5281/zenodo.6968695 [68].

## Abbreviations

VAB: Vibration attraction behavior
NOA: Number of approaches
DIR: Duration inside 1.3-cm radius
DOR: Duration outside 1.3-cm radius
SNs: Superficial neuromasts
IO3: 3Rd infraorbital bone
L–R: Left–right
DLC: DeepLabCut
