# Supplementary material for: Evolution of left–right asymmetry in the sensory system and foraging behavior during adaptation to food-sparse cave environments
Source: BMC Biol. 2022 Dec 27;20:295. doi: 10.1186/s12915-022-01501-1 (PMC9795734; doi:10.1186/s12915-022-01501-1)
Supplement: Supplementary file 1 — Additional file 1: Fig. S1. Correlation plots in SF. Fig. S2. Overall ablation plots in PA. Fig. S3. Left and Right NOA and DIR across populations. Fig. S4. Left and Right NOA and DIR before and after starvation. Fig.S5. Evolutionary model for sensory and behavior laterality. Table S1. Statistical Scores for Figs.1 and S3. Table S2. Correlation Scores for Fig. 1. Table S3. Correlation Scores for Fig. 2. Table S4. Statistical Scores for Fig.S2. Table S5. Statistical Scores for Figs.4 and S4. Table S6. Correlation Scores for Fig. 4. Table S7. Statistical Scores for Fig.5C and D. [file 12915_2022_1501_MOESM1_ESM.pdf]

Additional file 1
Supplementary Figures and Tables

Supplementary Figure 1

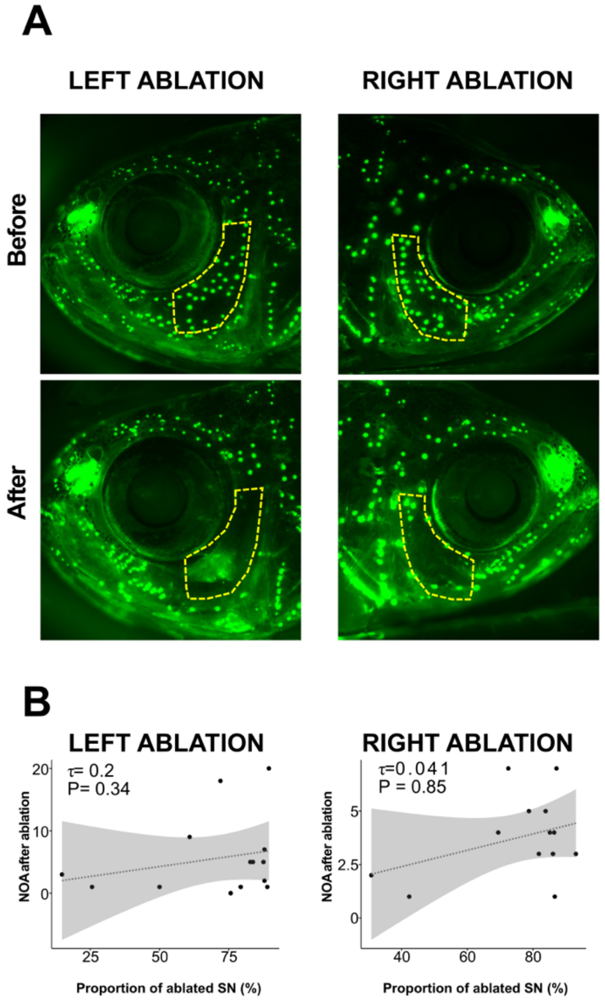

Figure S1. Ablation of superficial neuromasts (SNs) did not induce detectable effect on the level of VAB in surface fish.
(**A**) Left and right sides of SNs in Pachón cavefish stained with 4-Di-1-ASP (green dots) before and after ablation of SNs in the infraorbital region (IO3 shown by a yellow line) of the same fish. (**B**) Proportion of ablated SNs on the left and right sides plotted against the total NOA in both cases. Regression lines with 95% confidence intervals (shaded gray or orange) are shown in each panel. See Table S3 for additional details.

Supplementary Figure 2

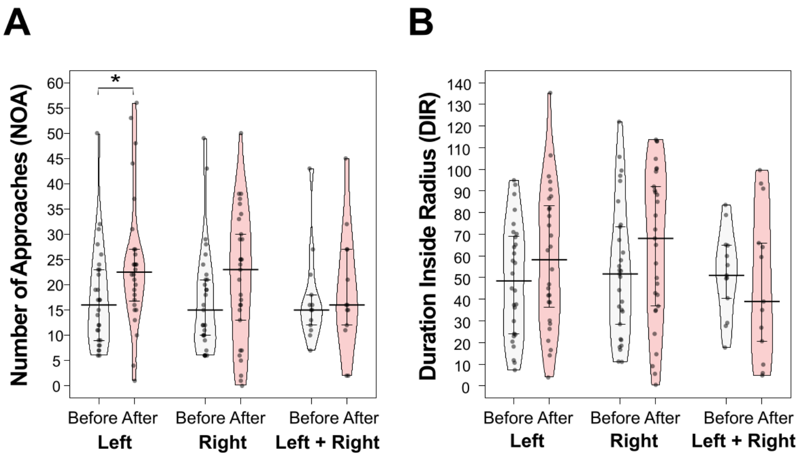

Figure S2. Overall results of the SN ablation experiments in Pachón cavefish.
(**A**) Number of approaches (NOA) in Pachón cavefish. Ablation of the left SN resulted in an increase in NOA (V = 74.5, P = 0.032); however, ablation of the right SN changed NOA at an underdetection level (V = 121, P = 0.063). (**B**) Duratin in the radius (DIR) in Pachón cavefish. Left SN or right SN ablation did not show detectable changes in DIR. Pirate plot bars represent means ± standard errors of the means. Data were analyzed using a generalized linear model followed by a post-hoc test (Wilcoxon test, adjusted by Holm's correction): *, P < 0.05; **, P < 0.01; and ***, P < 0.001. Statistical scores are available in Table S4.

Supplementary Figure 3

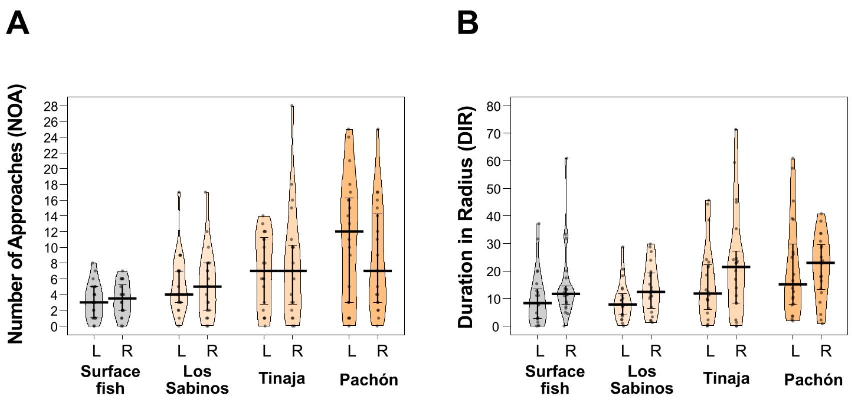

Figure S3. *Astyanax mexicanus* populations did not exhibit left- or right-biased approaches (NOA) or adhesion (DIR) at the population level.
(**A, B**) Pirate plots showing no detectable differences between the number of left- and right-side approaches (NOA) (**A**) or the duration in the radius (DIR) (**B**) among the four tested populations (2–3-year-old individuals). Data represent means ± standard errors of the means (each population, n = 20). Further details are presented in Table S1.

Supplementary Figure 4

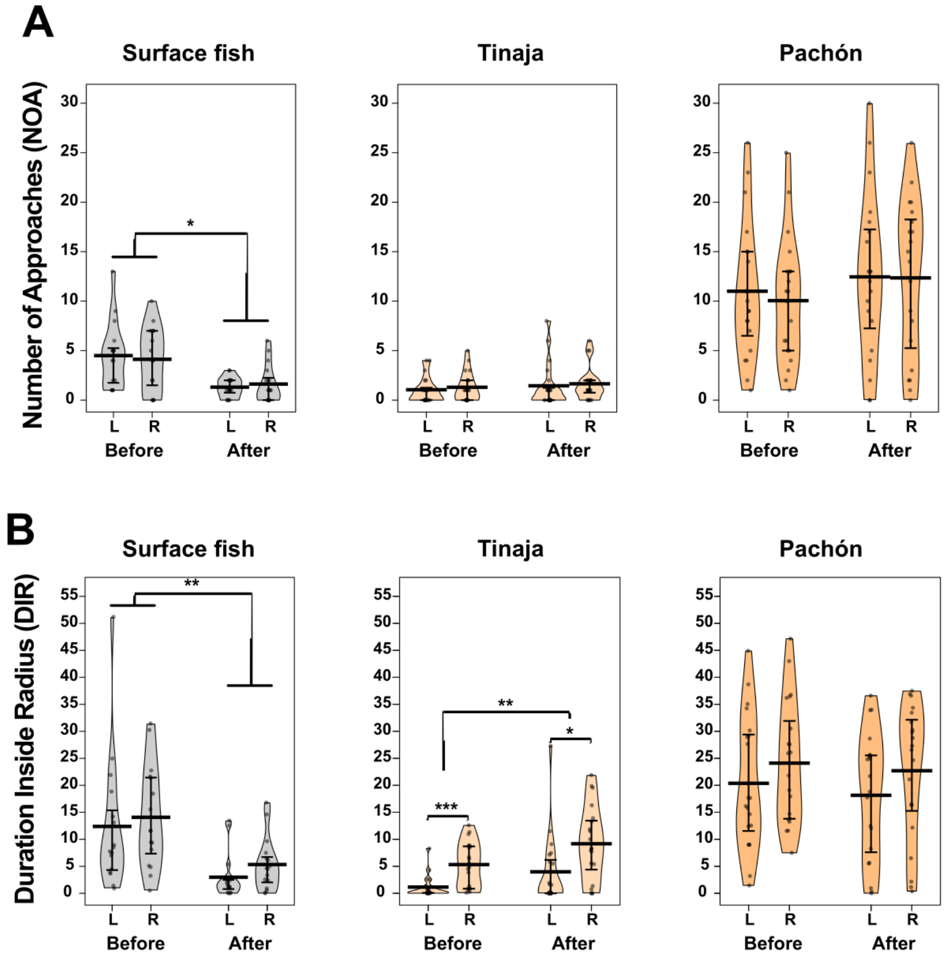

Figure S4. In response to starvation, lateralities in NOA and DIR are plastic in Tinaja cavefish but not in Pachón cavefish.

(**A, B**) Pirate plots showing the number of left- and right-side approaches (NOA) (**A**) and left- and right-side-associated durations within a 1.3-cm radius (DIR) (**B**) before and after a 6-day fasting period. In A, only surface fish showed a significantly reduced NOA after fasting among the three tested populations, and no L–R bias (laterality) was detectable. In **B**, surface fish showed significantly reduced DIR after fasting, whereas Tinaja cavefish showed increased DIR after fasting. Tinaja cavefish also showed right bias in DIR before and after fasting, but they showed more significant right bias than left bias in DIR before fasting. Pachón cavefish did not exhibit any changes in DIR or L–R bias in DIR. All statistical scores are available in Table S5. *, $P < 0.05$; **, $P < 0.01$.

Supplementary Figure 5

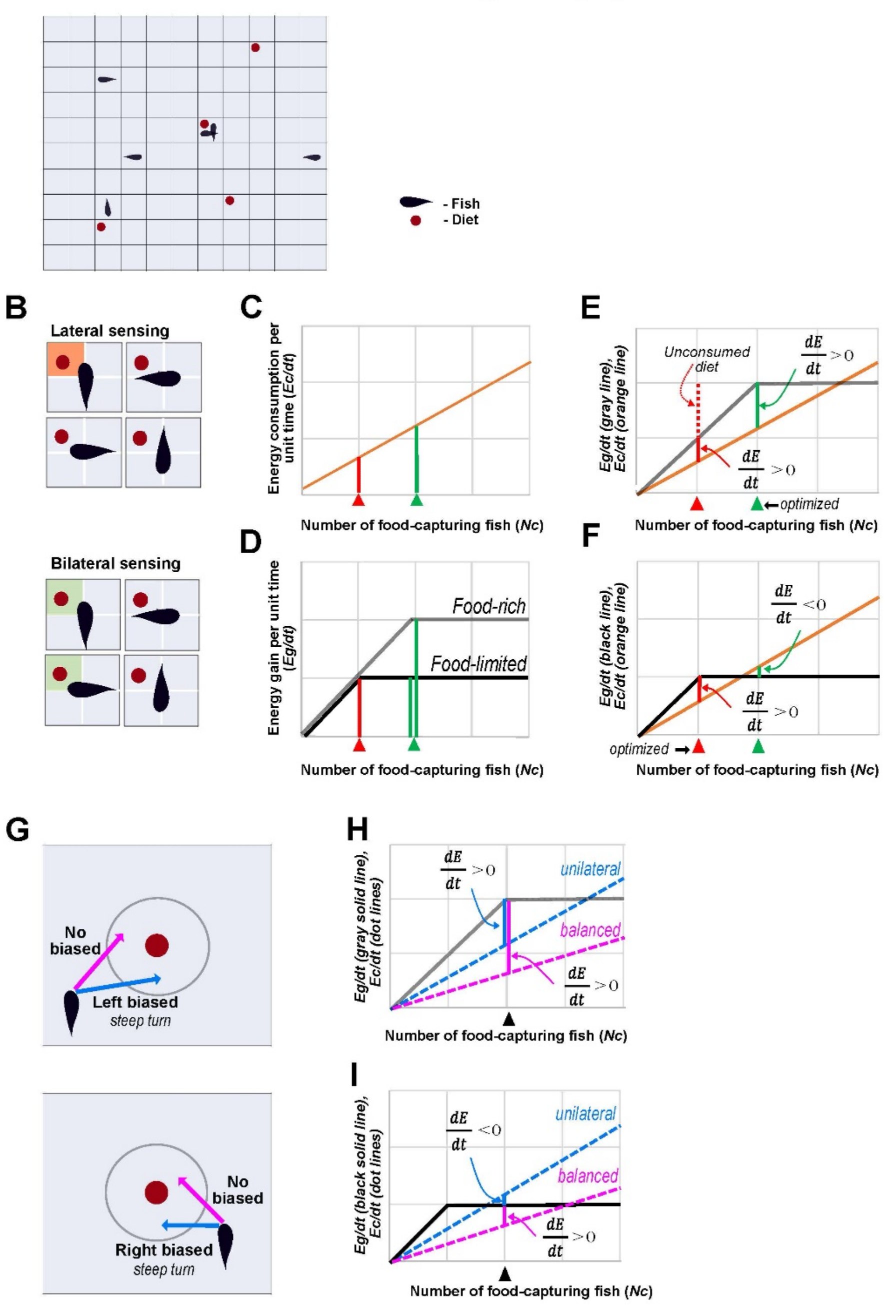

Figure S5. Relevance of sensory and behavior laterality.
(**A**) General schematic of the model showing food availability and the number of fish. Each cell represents the sensing range of a fish. Red dots represent diet and black shapes represent fish. (**B**) In a given cell, the food detection probability p_d is 1/4 for lateral sensing (top) and 1/2 for bilateral sensing (bottom); red and green shaded areas represent overlapping areas in the food and sensing fields, respectively. (**C**) Energy consumption per unit time ($\frac{dE_c}{dt}$) as a function of the number of food-acquiring fish (N_c). (**D**) Energy gain from food per unit time ($\frac{dE_g}{dt}$) as a function of the number of food-acquiring fish (N_c). In **C** and **D**, red arrows and lines indicate lateral sensing fish and green arrows and lines indicate bilateral sensing fish. Gray and black lines are the functions $\frac{dE_g}{dt}$ and depend on food-rich and -limited conditions, respectively. (**E, F**) Evaluation of $\frac{dE}{dt}$. E shows $\frac{dE_g}{dt}$ and $\frac{dE_c}{dt}$ under food-rich conditions, whereas **F** shows $\frac{dE_g}{dt}$ and $\frac{dE_c}{dt}$ under food-limited conditions. $\frac{dE}{dt}$ is based on the subtraction of $\frac{dE_c}{dt}$ from $\frac{dE_g}{dt}$ and is shown as solid vertical lines (green and red). In **E** (food-rich condition), the bilateral population is

optimized because $\frac{dE}{dt}$ of the bilateral sensing population is larger than that of the unilateral sensing population. Furthermore, there is no unconsumed food in the bilateral sensing population, whereas unconsumed food is present in the unilateral sensing population (red dotted line). In **F** (food-limited condition), the unilateral population is optimized because dE/dt of the unilateral sensing population has a positive value, whereas $\frac{dE}{dt}$ in the bilateral sensing population is negative. (**G**) Food-approaching behavior. The gray circle shows the 1.3-cm radius from the food (red circle). Blue and pink arrows represent steeper and less steep turns, respectively. Fish with balanced approaches take either left or right approaches, whereas fish with biased approaches (laterality) always take one-sided approaches that frequently require steeper turns. (**H, I**) Evaluation of $\frac{dE}{dt}$ in food-rich (**H**) and food-limited (**I**) environments. The $\frac{dE_c}{dt}$ values for unilateral (steeper turn) fish and balanced fish are shown as blue and pink dotted lines, respectively. $\frac{dE}{dt}$ is based on the subtraction of E_c (dotted lines) from $\frac{dE_g}{dt}$ (gray solid line in **H** or black in **I**); therefore, it is expressed as the blue and pink vertical solid lines. Under food-rich conditions, $\frac{dE}{dt}$ for both unilateral and balanced fish is positive; thus, both populations can gain energy. In contrast, under food-limited conditions, the L–R balanced population is optimal because it shows a positive value for $\frac{dE}{dt}$. See Additional file 2 for further information.

Table S1. Statistical scores for Figure 1 and Figure S3.

		Generalized linear model Statistical score	P-values	*Wilcoxon (pairwise comparison)*	P-values *(Holm's correction)*
**Swimming Distance**	Population (SF, LS, TI, PA)	F(3, 76) = 14.17	**2.00E-07*** **		
**Number of Approaches (NOA)**	Population (SF, LS, TI, PA)	F(3, 152)= 11.327	**9.52E-07*** **	SF vs LS	0.054
				SF vs TI	**0.004** **
	LR (Left, Right)	F(1, 152)= 0.149	0.700	SF vs PA	**7.7E-04*** **
				LS vs TI	0.232
	Pop × LR	F(3, 152)= 0.754	0.522	LS vs PA	0.053
				TI vs PA	0.232
**Duration Spent Inside the Radius (DIR)**	Population (SF, LS, TI, PA)	F(3, 152)= 5.098	**2.17E-03** **	SF vs LS	0.795
				SF vs TI	0.282
	LR (Left, Right)	F(1, 152)= 3.378	0.068	SF vs PA	**0.026***
				LS vs TI	0.282
	Pop × LR	F(3, 152)= 0.498	0.684	LS vs PA	**0.016***
				TI vs PA	0.620
**Duration Spent Outside the Radius (DOR)**	Population (SF, LS, TI, PA)	F(3, 152)= 0.861	0.463		
	LR (Left, Right)	F(1, 152)= 0.560	0.455		
	Pop x LR	F(3, 152)= 0.016	0.997		

Surface fish (SF, n = 20) and Los Sabinos (LS, n = 20), Tinaja (TI, n = 20), and Pachón (PA, n = 20) cavefish.
Linear or generalized linear models were selected using Akaike's information criterion function to determine the best fit model for analyzing swimming distance, NOA, DIR, and DOR.
***: Significant at alpha = 0.001; **: significant at alpha = 0.01; *: significant at alpha = 0.05.

Table S2. Correlation analysis for Figure 1.

	SURFACE FISH		LOS SABINOS		TINAJA		PACHÓN	
	**Kendall Tau**	**P-value**	**Kendall Tau**	**P-value**	**Kendall Tau**	**P-value**	**Kendall Tau**	**P-value**
**Correlation against total NOA**								
Left SN number	−0.145	0.420	−0.147	0.377	−0.145	0.379	**0.329**	**0.047***
Right SN number	−0.158	0.378	−0.264	0.116	−0.048	0.770	0.258	0.118
Total SN number	−0.130	0.466	−0.201	0.227	−0.145	0.379	0.269	0.103
**Correlation against total DIR**								
Left SN number	−0.238	0.172	−0.027	0.871	−0.095	0.559	0.292	0.074
Right SN number	−0.223	0.198	−0.053	0.745	0.106	0.516	0.212	0.194
Total SN number	−0.223	0.198	−0.058	0.721	−0.053	0.745	0.265	0.104
**Correlation against left NOA**								
Left SN number	−0.272	0.133	−0.185	0.277	0.125	0.452	0.312	0.059
Right SN number	**−0.388**	**0.031***	−0.175	0.306	0.267	0.109	0.263	0.110
**Correlation against right NOA**								
Left SN number	0.070	0.699	−0.141	0.396	−0.243	0.142	0.269	0.103
Right SN number	0.098	0.589	−0.225	0.180	−0.102	0.536	0.241	0.143
**Correlation against left DIR**								
Left SN number	−0.277	0.111	0.000	1.000	−0.011	0.948	**0.324**	**0.047***
Right SN number	**−0.341**	**0.049***	0.027	0.871	0.254	0.119	0.201	0.217
**Correlation against right DIR**								
Left SN number	−0.185	0.288	−0.090	0.581	−0.133	0.417	0.245	0.135
Right SN number	−0.144	0.404	−0.086	0.602	−0.037	0.820	0.164	0.314

ANOVA was performed using the generalized linear model fitting function. Population differences for NOA and DIR were determined via pairwise comparisons using a Wilcoxon rank sum test with continuity correction. Holm's correction method was used for P-value adjustment.
***: Significant at alpha = 0.001; **: significant at alpha = 0.01; *: significant at alpha = 0.05.

Table S3. Correlation analysis for Figure 2.

	Surface fish		Pachón cavefish (number of approaches > 6)	
	**Kendall Tau R**	**P-value**	**Kendall Tau R**	**P-value**
**Correlation against Total NOA after ablation**				
Left SN ablation (%)	0.198	0.342	**−0.342**	**0.009****
Right SN ablation (%)	0.041	0.852	−0.064	0.635
Left + Right SN ablation (%)	0.159	0.528	−0.237	0.269
**Correlation against Total DIR after ablation**				
Left SN ablation (%)	0.155	0.443	−0.094	0.479
Right SN ablation (%)	0.179	0.435	0.003	0.984
Left + Right SN ablation (%)	0.289	0.291	−0.026	0.952
**Correlation against Left NOA after ablation**				
Left SN ablation (%)	0.189	0.383	−0.376	**0.004****
Right SN ablation (%)	0.042	0.850	−0.032	0.812
**Correlation against Right NOA after ablation**				
Left SN ablation (%)	0.070	0.753	−0.057	0.677
Right SN ablation (%)	0.128	0.539	−0.189	0.148
**Correlation against Left DIR after ablation**				
Left SN ablation (%)	0.287	0.154	−0.147	0.254
Right SN ablation (%)	0.116	0.582	−0.098	0.465
**Correlation against Right DIR after ablation**				
Left SN ablation (%)	−0.013	0.951	0.056	0.678
Right SN ablation (%)	0.122	0.546	−0.085	0.524

***: Significant at alpha = 0.001; **: significant at alpha = 0.01; *: significant at alpha = 0.05.
Left SN: superficial neuromasts in the left-side; Right SN: superficial neuromasts in the right-side; Left + Right SN: sum of the number of superficial neuromasts present in both left and right sides; NOA: number of approaches; DIR: duration inside a 1.3-cm radius from the rod; Left NOA: number of approaches to the rod with the left-side of the head; Right NOA: number of approaches to the rod with the right-side of the head; Left DIR: duration inside a 1.3-cm radius from the rod with left-side head facing the rod; Right DIR: duration inside a 1.3-cm radius from the rod with right-side head facing the rod; Total NOA: sum of Left NOA and Right NOA; Total DIR: Sum of Left NOA and Right DIR.
Only Kendall Tau R values that satisfy P < 0.05 with 95% confidence intervals that do not cross 0 are shown in bold.

Table S4. Statistical scores for Figure S2.

	ANOVA			*Wilcoxon signed-rank (paired)*			*Holm's correction*	
		**statistics**	**p-value**		**statistics**	**p-value**	**alpha = 0.05**	**K = 3**
**Swimming Distance**	Ablation	$\chi^2(2)$= 0.7	0.965					
(glm)	Prepost	$\chi^2(1)$= 1.3	0.245					
	Ablation:Prepost	$\chi^2(2)$= 4.8	0.091					
**Number Of Approaches**	Ablation	$\chi^2(2)$= 1.7	0.431	PA Left (Pre vs. Post)	V = 74.5	0.011	**0.032***	
**(NOA)**	Prepost	$\chi^2(1)$= 8.8	**0.003****	PA Right (Pre vs. Post)	V = 121	0.063		
**(glm)**	LR	$\chi^2(1)$= 0.1	0.713	PA Left+Right (Pre vs. Post)	V = 39	1		
	Ablation:Prepost	$\chi^2(2)$= 1.1	0.586					
	Ablation:LR	$\chi^2(2)$= 0.1	0.973					
	Prepost:LR	$\chi^2(1)$= 0.5	0.479					
	Ablation:Prepost:LR	$\chi^2(2)$= 0.9	0.643					
**Duration Spent Inside**	Ablation	$\chi^2(2)$= 2.6	0.276	PA Left (Pre vs. Post)	V = 139	0.150		
**The Radius (DIR)**	Prepost	$\chi^2(1)$= 4.3	**0.039***	PA Right (Pre vs. Post)	V = 138	0.088		
**(lmer)**	LR	$\chi^2(1)$= 0.0	0.964	PA Left+Right (Pre vs. Post)	V = 50	0.787		
	Ablation:Prepost	$\chi^2(2)$= 2.4	0.306					
	Ablation:LR	$\chi^2(2)$= 0.3	0.858					
	Prepost:LR	$\chi^2(1)$= 0.4	0.506					
	Ablation:Prepost:LR	$\chi^2(2)$= 0.1	0.950					
**Duration Spent Outside**	Ablation	$\chi^2(2)$= 0.4	0.800					
**The Radius (DOR)**	Prepost	$\chi^2(1)$= 1.6	0.202					
**(lmer)**	LR	$\chi^2(1)$= 3.6	0.058					
	Ablation:Prepost	$\chi^2(2)$= 2.7	0.256					
	Ablation:LR	$\chi^2(2)$= 4.1	0.127					
	Prepost:LR	$\chi^2(1)$= 0.2	0.648					
	Ablation:Prepost:LR	$\chi^2(2)$= 1.0	0.621					

Left (PA, n = 28), right (PA, n = 29), and both left and right (PA, n = 13) sides.
Linear or generalized linear models were selected using Akaike's information criterion function to determine the best fit model for analyzing swimming distance, NOA, DIR, and DOR.
***: Significant at alpha = 0.001; **: significant at alpha = 0.01; *: significant at alpha = 0.05.
ANOVA was performed using the generalized linear models fitting function. Post-hoc tests were performed using the Wilcoxon signed-rank (paired) test followed by Holm's multiple-test correction.

Table S5. Statistical scores for Figure 4 and Figure S4.

	**Generalized linear model**			**Wilcoxon signed-rank (paired)**			**Holm's correction**	
		Statistics	p-value		statistics	p-value	alpha = 0.05	
Swimming Distance	Population (SF, TI, PA)	F(2, 106) = 67.214	< 2E-16	Before vs. After (SF)	V = 37	0.117	0.233	K= 3
	Starvation (Before, After)	F(1, 106) = 7.417	7.56E-03	Before vs. After (TI)	V = 207	9.54E-06	**2.86E-05*** **	
	Pop:Starvation	F(2, 106) = 10.199	8.89E-05	Before vs. After (PA)	V = 106	0.985	0.985	
Number of Approaches (NOA)	LR (Left, Right)	F(1, 212) = 0.033	0.855	Before vs. After (SF)	V = 8.5	3.74E-03	**0.011***	K= 3
	Population (SF, TI, PA)	F(2, 212) = 102.928	<2E-16	Before vs. After (TI)	V = 81	0.846	0.846	
	Starvation (Before, After)	F(1, 212) = 0.000	0.989	Before vs. After (PA)	V = 134	0.286	0.573	
	LR:Pop	F(2, 212) = 0.128	0.880					
	LR:Starvation	F(1, 212) = 0.144	0.705					
	Pop:Starvation	F(2, 212) = 4.485	0.012					
	LR:Pop:Starvation	F(2, 212) = 0.050	0.951					
Duration Spent Inside the Radius (DIR)	LR (Left, Right)	F(1, 212) = 9.469	2.37E-03	Before vs. After (SF)	V = 7	1.76E-03	**5.27E-03****	K= 3
	Population (SF, TI, PA)	F(2, 212) = 71.014	<2E-16	Before vs. After (TI)	V = 183	2.33E-03	**4.65E-03****	
	Starvation (Before, After)	F(1, 212) = 2.856	0.092	Before vs. After (PA)	V = 87	0.522	0.522	
	LR:Pop	F(2, 212) = 0.415	0.661	L vs. R (SF, Before)	V = 39	0.144	0.144	K= 6
	LR:Starvation	F(1, 212) = 0.122	0.728	L vs. R (SF, After)	V = 20	0.025	0.099	
	Pop:Starvation	F(2, 212) = 8.340	3.26E-04	L vs. R (TI, Before)	V = 11	1.05E-04	**6.29E-04*** **	
	LR:Pop:Starvation	F(2, 212) = 0.002	0.998	L vs. R (TI, After)	V = 13	2.86E-03	**0.014***	
				L vs. R (PA, Before)	V = 48	0.035	0.105	
				L vs. R (PA, After)	V = 52	0.048	0.097	
Duration Spent Outside the Radius (DOR)	LR (Left, Right)	F(1, 212) = 7.281	7.53E-03	Before vs. After (SF)	V = 131	3.05E-04	**9.16E-04*** **	K= 3
	Population (SF, TI, PA)	F(2, 212) = 5.405	5.14E-03	Before vs. After (TI)	V = 69	0.185	0.370	
	Starvation (Before, After)	F(1, 212) = 0.379	0.539	Before vs. After (PA)	V = 124	0.498	0.498	
	LR:Pop	F(2, 212) = 5.269	5.84E-03	L vs. R (SF, Before)	V = 63	0.821		
	LR:Starvation	F(1, 212) = 0.264	0.608	L vs. R (SF, After)	V = 82	0.495		
	Pop:Starvation	F(2, 212) = 0.634	0.531	L vs. R (TI, Before)	V = 65	0.143		
	LR:Pop:Starvation	F(2, 212) = 0.237	0.789	L vs. R (TI, After)	V = 71	0.216		
				L vs. R (PA, Before)	V = 84	0.452		
				L vs. R (PA, After)	V = 95	0.729		

Surface fish (SF, n = 16),Tinaja cavefish (TI, n = 20), and Pachón cavefish (PA, n = 20).
Linear or generalized linear models were selected using Akaike's information criterion function to determine the best fit model for analyzing swimming distance, NOA, DIR, and DOR.
***: Significant at alpha = 0.001; **: significant at alpha = 0.01; *: significant at alpha = 0.05.
ANOVA was performed using the generalized linear model fitting function. Post-hoc tests were performed using the Wilcoxon signed-rank (paired) test followed by Holm's multiple-test correction.

Table S6. Correlation analysis for Figure 4.

	SURFACE FISH		TINAJA		PACHÓN	
	**Kendall Tau**	**P-value**	**Kendall Tau**	**P-value**	**Kendall Tau**	**P-value**
**Correlation against total NOA (Before)**						
Left SN number	0.226	0.236	0.053	0.762	0.226	0.182
Right SN number	0.175	0.361	-0.100	0.568	0.173	0.323
**Correlation against total NOA (After)**						
Left SN number	−0.379	0.052	0.267	0.124	−0.030	0.861
Right SN number	**−3.292**	**9.96E-04*** **	0.104	0.548	−0.113	0.518
**Correlation against increased amounts of total NOA (After - Before)**						
Left SN number	−0.315	0.094	0.240	0.163	**−0.394**	**0.020***
Right SN number	−0.352	0.063	0.182	0.288	−0.340	0.052

***: Significant at alpha = 0.001
**: Significant at alpha = 0.01
*: Significant at alpha = 0.05

Table S7. Statistical scores for Figure 5C and D.

	**General Mixed-Effects Mode (Gamma, Random effect = individual fish)**			**General Mixed-Effects Mode (Gamma,Random effect = individual fish)**			**Holms correction**	
		Statistics			statistics	p-value	alfa = 0.05	
Bias Index (NOA)	Population (SF, TI, PA)	$\chi^2(2)$= 1.667	0.436	Before vs After (SF)	$\chi^2(1)$= 0.011	0.917		K= 3
	Starvation (Before, After)	$\chi^2(1)$= 0.300	0.584	Before vs After (TI)	$\chi^2(1)$= 10.278	1.35E-03	**4.04E-03** **	
	Pop:Starvation	$\chi^2(2)$= 7.532	0.023	Before vs After (PA)	$\chi^2(1)$= 0.267	0.605		
Bias Index (NOA)	Population (SF, TI, PA)	$\chi^2(2)$= 10.945	0.042	Before vs After (SF)	$\chi^2(1)$= 0.006	0.936		K= 3
	Starvation (Before, After)	$\chi^2(1)$= 0.540	0.462	Before vs After (TI)	$\chi^2(1)$= 5.245	0.022	0.066	
	Pop:Starvation	$\chi^2(2)$= 5.182	0.075	Before vs After (PA)	$\chi^2(1)$= 0.198	0.656		

Surface fish (SF, n= 17),Tinaja (TI, n= 9), and Pachón (PA, n= 20).
Linear or generalized linear models were selected using the Akaike's information criterion function to address the best fit model to analyze bias index for NOA and DIR.
***: Significant at alpha = 0.001
**: Significant at alpha = 0.01
*: Significant at alpha = 0.05
Analysis of variance was done with the General Mixed-Effects Model fitting function. Post-hoc tests were performed using the General Mixed-Effects Model by Holm's multiple-test correction.
