# Supplementary material for: Evolution of left–right asymmetry in the sensory system and foraging behavior during adaptation to food-sparse cave environments
Source: BMC Biol. 2022 Dec 27;20:295. doi: 10.1186/s12915-022-01501-1 (PMC9795734; doi:10.1186/s12915-022-01501-1)
Supplement: Supplementary file 2 — Additional file 2. Extended results and discussion. [file 12915_2022_1501_MOESM2_ESM.pdf]

Additional file 2
Extended results and discussion

VAB of Tinaja cavefish was higher than that of Los Sabinos cavefish, contradicting results reported previously.
In Figure 1C, Tinaja cavefish are shown to have a similar VAB level to that of Pachón cavefish, both in terms of NOA (Pachón vs. Tinaja: P = 0.620) and DIR (Pachón vs. Tinaja: P = 0.232) (also see Supplementary Table 1). Contrastingly, the VAB level of Los Sabinos cavefish (both NOA and DIR) was indistinguishable from that of surface fish (surface fish vs. Los Sabinos: NOA, P = 0.054; DIR, P = 0.795; Figure 1C and Supplementary Table 1). In a previous study, Los Sabinos cavefish showed a higher VAB level than that of Tinaja cavefish (1). Thus, VAB may be plastic in Tinaja and Los Sabinos cavefish. Indeed, we detected a plastic change in VAB in Tinaja cavefish according to food availability and age (see the main text). Interestingly, Tinaja and Los Sabinos caves are close geographically and thought to be connected (2).

Increase in NOA after SN ablation in Pachón cavefish
In Pachón cavefish, ablation of the left SNs (but not right SNs or left and right SNS) induced an increase in NOA (Supplementary Figure 1). The increase in NOA after SN ablation contradicted the findings of previous studies in which SN ablation (both left and right SNs) reduced NOA (3,4). These discrepant results may be due to the following: (i) the selection of Pachón cavefish individuals with high VAB levels (NOA ≥ 6; see Materials and Methods) for ablation in the present study, potentially including individuals with high appetite/internal motivation by default; (ii) the use of an SN ablation area in the IO3 area that was not sufficiently large at this stage in the fish (i.e., a larger SN ablation area in the trunk reduces NOA (3); (iii) an inflammatory reaction after injury that enhanced sensitivity (5,6); and (iv) individual Pachón cavefish with fewer left SNs showing plasticity in NOA (increased NOA) in response to fasting (Figure 4C). In the current study, we did not test any of these possibilities; however, considering that one or more of these explanations is accurate, the tested fish might express a higher response to the vibration stimulus even with the loss of SNs. These possibilities should be investigated further in subsequent research.

Laterality in Tinaja cavefish according to age
In the lab, 1-year-old Tinaja cavefish showed a right-side bias in DIR before and after starvation (Supplementary Figure 4A). Thus, 1-year-old Tinaja cavefish with right-side bias showed plasticity in DIR in response to food availability. In addition, the degrees of L–R bias in NOA and DIR were also plastic and became symmetrical after starvation (Figure 5A, B; Supplementary Figure 4).
Interestingly, the right-side bias found in 1-year-old Tinaja cavefish was not detected in 2–3-year-old Tinaja cavefish. Notably, the overall VAB level (NOA and DIR) observed in 1-year-old Tinaja cavefish was lower than that in 2–3-year-old Tinaja cavefish (Supplementary Figure 3; and Supplementary Figure 4). Previous studies have shown that VAB level depends on age in Pachón cavefish (3,4) and varies among pools within the same cave (Pachón, Tinaja, and Molino caves) (7). Considering these findings, L–R bias in NOA and DIR and the overall levels of NOA and DIR could depend on environmental conditions and developmental stage. However, given standardized conditions, these L–R biases and VAB levels are consistent in the same individuals' ((3); also see the main text).
In summary, the L–R bias in VAB and overall VAB levels can vary even within the same cave population. Among the four populations tested here, Tinaja cavefish were the most plastic in terms of VAB level and L–R bias.

Relevance of sensory and behavior laterality in terms of calorie gain and consumption

1. Sensory laterality

We hypothesized that sensory laterality is beneficial in a food-limited environment in terms of the tradeoff between calorie gain and calorie consumption at the population level.

The change in the total amount of calories held in a population per unit time can be described as follows:

$$\frac{dE}{dt} = \frac{dE_g}{dt} - \frac{dE_c}{dt} \quad (1)$$

where E is total calories (energy), t is time, E_g is the calorie gain due to food uptake, and E_c is the calorie consumption per population due to food capturing (foraging). Compared with the typical swimming motion, food capturing motions are assumed to consume more calories because they involve quick turns toward the food item (personal observation of how the studied fish acquire food aggressively). When $\frac{dE}{dt} > 0$, the population can survive in a given environment; when $\frac{dE}{dt} < 0$, the number of individuals in a population will decline.

We expect that sensory laterality is a key property affecting the tradeoff between $\frac{dE_g}{dt}$ and $\frac{dE_c}{dt}$.

To deliver $\frac{dE_g}{dt}$ and $\frac{dE_c}{dt}$, we assume the following conditions:

Assumption	Description
**1**	A small cell area is defined as the range area in which the fish can sense food (Supplemental Figure 5A). The whole area is defined as *A,* i.e., the number of cell areas in the whole area.
**2**	The amount of food supplied in a unit of time in a field (denoted as F) is constant, and in the next time step the food is gone, i.e., the number of food items in a field is always same.
**3**	Food items are randomly dispersed in a field; however, for simplicity, food items are always in the top left corner in a cell area.
**4**	Fish exist in random positions and directions in an entire field. For simplicity, fish are positioned at the center of the cell area.
**5**	The number of food items captured by a fish is set as 1 (constant) when the food is in the same cell area. Even if there are two food items in the same cell area, the fish can only acquire one food item at a time.

Thus, total calorie consumption in a population per unit time, $\frac{dE_c}{dt}$, is given as follows:

$$\frac{dE_c}{dt} = a \times A \times p_f \times N_c, \quad (2)$$

where a is the coefficient of the average calorie consumption per fish to reach the food in a cell, A is the number of cells in the whole field, p_f is the probability that food exists in a cell area, and N_c is the number of fish engaging in food capturing in a cell area. Additionally, p_f is calculated as follows:

$$p_f = \begin{cases} F/A \text{ } if \text{ } F < A \\ 1 \text{ } if \text{ } F \geq A \end{cases}. \quad (3)$$

N_c is calculated as follows:

$$N_c = p_d \times \frac{N}{A}, \quad (4)$$

where p_d is the probability of sensing the food when the food exists in a cell area. Depending on whether VAB is regulated by the unilateral or bilateral sensory input, the value of p_d is defined as shown in Supplemental Figure 5B.

$$p_d = \begin{cases} 1/4\ for\ lateral\ sensing \\ 1/2\ for\ bilateral\ sensing \end{cases}. \tag{5}$$

Note that the N_c for unilateral sensing is half that for bilateral sensing. When only one food item in the cell area is available but two fish exist in the same cell area, the following apply: (i) in case of bilateral sensing, the two fish compete for one food item, resulting in one fish losing calories by failing to capture the food; (ii) for unilateral sensing, only one fish senses the food and acquires it, whereas the other fish does not evoke VAB and loses no calories.

As shown in eq. (2), $\frac{dE_c}{dt}$ is a linear function of N_c (Supplemental Figure 5C). Because the N_c for bilateral sensing is twice that for unilateral sensing, the $\frac{dE_c}{dt}$for bilateral sensing is also twice as high as that for unilateral sensing. Again, when multiple fish compete for limited food, we expect that the unilateral sensing population will save relatively more calories; therefore, such sensing can be considered adaptive to the environment because of reduced calorie consumption ($\frac{dE_c}{dt}$).

Total calorie gain in a population by food uptake per unit time, $\frac{dE_g}{dt}$, is defined as follows:

$$\frac{dE_g}{dt} = b \times A \times F_C, \tag{6}$$

where b is the coefficient of the efficacy of calorie gain from a food item and F_c is the number of food items consumed in a cell area. F_c is a function of N_c. If there are sufficient food items, F_c increases linearly depending on how many fish are in a cell area. When food is limited, F_c is saturated to the upper limit of food items in a cell. Thus, F_c can be expressed using the following equations based on the assumption (Assumption 5) stated above (the number of food items a fish can consume per unit time dt is 1):

$$F_c = \begin{cases} F/A \times N_c\ \ if\ F/A > N_c \\ F/A\ if\ F/A \leq N_c \end{cases}. \tag{7}$$

In eqs. (6) and (7), $\frac{dE_g}{dt}$ is expressed as a function of N_c in Supplemental Figure 5D. As shown in Supplemental Figure 5D, $\frac{dE_g}{dt}$ can differ depending on the food supply in an area. If the number of food items is large, $\frac{dE_g}{dt}$ has a linear relationship with N_c even if the value of N_c is large, but if the number of food items is small, E_g is saturated to the upper limit.

Finally, $\frac{dE}{dt}$ in eq. (1) can be rewritten with eqs. (2) and (6) as a function of N_c as follows:

$$\frac{dE(N_c)}{dt} = b \times A \times F_c(N_c) - a \times A \times p_f \times N_c, \tag{8}$$

where the parameters $a, b, A,$ and F have the following condition:

$$b \times F > a \times A. \quad (9)$$

Thus, eq. (8) must output a positive value at a certain domain, otherwise $\frac{dE(N_c)}{dt}$ is negative for every N_c and the population size decreases.

Equation (8) can be examined by overlaying the graphs shown in Supplemental Figure 5C and D, as shown in Supplemental Figure 5E and F. $\frac{dE(N_c)}{dt}$ differs depending on whether the fish are in a food-rich or -limited environment. Under food-rich conditions, the bilateral sensing population is optimized because $\frac{dE(N_c)}{dt}$ is larger than that in the lateral sensing population (Supplemental Figure 5E). Under food-limited conditions, however, unilateral sensing is optimized because $\frac{dE(N_c)}{dt}$ is positive, whereas that for bilateral sensing is negative (Supplemental Figure 5F). In the Pachón cave, food is known to be limited (c.f., ref (8)); therefore, lateral sensing is expected to be optimal in the Pachón environment.

2. Laterality in approaches (behavior laterality)

Behavior laterality is defined as foraging approaches made dominantly from one side (left or right) after VAB is triggered in the fish. From video observations, fish were found to make sharp turns toward the vibrating rod when approaching in the vicinity of the rod (within 1.3 cm). We predict that sharp turning consumes more calories than typical swimming. We also assume that fish that show unilateral approaches turn with steeper angles compared with fish approaching the food from both sides. This assumption is based on the notion that the position of the fish relative to the food is stochastic (Supplemental Figure 5G).

Here, the larger energy loss in the biased approaches is expressed as $\frac{dE_c}{dt}$ in eq. (1). Specifically, the difference between the unilateral or balanced approaches is expressed by the parameter a, the coefficient of the average calorie consumption per fish. Accordingly, a has a higher value in fish that make unilateral approaches relative to those that make balanced approaches. These scenarios are shown in Supplemental Figure 5H and 5I.

The value of $\frac{dE(N_c)}{dt}$ differs depending on whether conditions are food-rich or -limited. As shown in Supplemental Figure 5H, $\frac{dE(N_c)}{dt}$ is positive for fish that make unilateral and balanced approaches under food-rich conditions; thus, both populations will gain energy. In contrast, under food-limited conditions, making balanced approaches is optimal because $\frac{dE(N_c)}{dt}$ has a positive value (Supplemental Figure 5I). Our fasting experiment showed that Tinaja cavefish shifted their approach from unilateral to balanced; therefore, these cavefish are able to adjust their foraging behavior depending on food availability. The sensory-foraging behavior of Pachón cavefish is further optimized to food-limited conditions in terms of both sensing food (sensory unilaterality) and foraging behavior (balanced approach) because both unilateral sensing and balanced approaches can reduce wasting energy.

Supplementary references

1. Yoshizawa M, Robinson BG, Duboué ER, Masek P, Jaggard JB, O'Quin KE, et al. Distinct genetic architecture underlies the emergence of sleep loss and prey-seeking behavior in the Mexican cavefish. BMC Biol. 2015;13(1):15.
2. Mitchell RW, Russell WH, Elliott WR. Mexican eyeless characin fishes, genus Astyanax: Environment, distribution, and evolution. Special pu. Mackey C, Barnett GE, editors. Special publications the museum Texas Tech University. Texas: Texas Tech Press; 1977. 89 p.
3. Yoshizawa M, Gorički S, Soares D, Jeffery WR. Evolution of a behavioral shift mediated by superficial neuromasts helps cavefish find food in darkness. Curr Biol. 2010;20(18):1631–6.
4. Yoshizawa M, Yamamoto Y, O'Quin KE, Jeffery WR. Evolution of an adaptive behavior and its sensory receptors promotes eye regression in blind cavefish. BMC Biol. 2012;10(1):108.
5. Neumann S, Doubell TP, Leslie T, Woolf CJ. Inflammatory pain hypersensitivity mediated by phenotypic switch in myelinated primary sensory neurons. Nature. 1996;384(6607):360–4.
6. Wang H, Dai Y, Fukuoka T, Yamanaka H, Obata K, Tokunaga A, et al. Enhancement of stimulation-induced ERK activation in the spinal dorsal horn and gracile nucleus neurons in rats with peripheral nerve injury. Eur J Neurosci. 2004;19(4):884–90.
7. Espinasa L, Heintz C, Rétaux S, Yoshisawa M, Agnès F, Ornelas-Garcia P, et al. Vibration attraction response is a plastic trait in blind Mexican tetra (*Astyanax mexicanus*), variable within subpopulations inhabiting the same cave. J Fish Biol. 2021 Oct 30;98(1):304–16.
8. Espinasa L, Bonaroti N, Wong J, Pottin K, Queinnec E, Rétaux S. Contrasting feeding habits of post-larval and adult *Astyanax* cavefish. Subterr Biol. 2017;21:1–17.
